# Polymerization shrinkage behaviour of resin composites functionalized with unsilanized bioactive glass fillers

**DOI:** 10.1038/s41598-020-72254-6

**Published:** 2020-09-17

**Authors:** Matej Par, Dirk Mohn, Thomas Attin, Zrinka Tarle, Tobias T. Tauböck

**Affiliations:** 1 Department of Conservative and Preventive Dentistry, Centre for Dental Medicine, University of Zurich, Plattenstrasse 11, Zurich, Switzerland; 2 Department of Endodontics and Restorative Dentistry, School of Dental Medicine, University of Zagreb, Gunduliceva 5, Zagreb, Croatia; 3 Department of Chemistry and Applied Biosciences, Institute for Chemical and Bioengineering, ETH Zurich, Zurich, Switzerland

**Keywords:** Composite resin, Dental biomaterials

## Abstract

Previous work has shown that partial replacement of reinforcing fillers with unsilanized silica particles can diminish polymerization shrinkage stress of dental resin composites. The aim of the present study was to investigate whether such an effect can be attained by using unsilanized bioactive glass (BG). Incorporating BG fillers into resin composites is interesting due to their potential for exerting caries-preventive effects. Experimental light-curable composites with a total filler load of 77 wt% were prepared. Reinforcing fillers were partially replaced with 0–60 wt% of BG 45S5 and an experimental low-sodium fluoride-containing BG. The following properties were investigated: linear shrinkage, degree of conversion, shrinkage stress, maximum shrinkage stress rate, and time to achieve maximum shrinkage stress rate. The diminishing effect of BG 45S5 on shrinkage stress was mediated by a decrease in degree of conversion caused by this BG type. In contrast, as the degree of conversion remained unaffected by the experimental BG, the resulting shrinkage behaviour was governed by the effect of varying amounts of silanized and unsilanized fillers on material’s viscoelastic properties. The replacement of silanized reinforcing fillers with unsilanized BG did not reduce polymerization shrinkage stress unless the reduction was attained indirectly through a diminished degree of conversion.

## Introduction

The persistent issue of secondary caries around composite restorations makes the research on bioactive resin composites with anti-caries activity a logical next step in the development of restorative composites^1^. Bioactive composites functionalized with various types of reactive fillers have been extensively investigated due to their ion-releasing^2^, antibacterial^3^, alkalizing^4^, and calcium phosphate-forming capabilities^5,6^. An especially interesting type of reactive filler capable of exerting all of these beneficial effects pertains to the group of bioactive glasses (BGs). These reactive glasses with adjustable composition and properties have shown a promising potential to render resin composites more resistant to secondary caries^5,7–12^. A particularly interesting modification of BGs involves fine-tuning of their sodium amount to attain a prolonged bioactive effect, and introducing fluorides as therapeutic ions capable of remineralizing dental hard tissues^5,7,12^. The inevitable shortcoming of reactive BG fillers is their detrimental effect on the mechanical properties of the composite, especially when used in higher amounts^13,14^. However, this negative effect can be mitigated by adjusting the ratio of BG and reinforcing fillers to the level which is sufficient to produce a desired bioactive effect, and yet does not significantly impair mechanical properties^15^.

Dental resin composites are composed of inorganic filler particles dispersed in a photo-curable resinous matrix. The filler particles are commonly coated with a functional silane, which enables their chemical bonding to the polymeric matrix^16^. The Si–O–Si bonds at the filler/matrix interface enhance the distribution of mechanical loads between the two phases with considerably different elastic moduli, thereby reinforcing the composite’s structure and improving its resistance to fracture^17^. Silane coating of filler particles has been used since the inception of resin composites, and its basic chemistry remained unchanged up to date. However, in bioactive composites, reactive fillers are preferably used without surface silanization^2–5,7–12,18^. The hydrophobic silane coating on reactive fillers is undesired as their surfaces must remain accessible to water in order to be able to dissolve and exert bioactive effects^19^. Therefore, experimental bioactive composites commonly contain varying ratios of two filler types: (I) non-soluble silanized fillers, which reinforce the material structure; and (II) reactive unsilanized fillers, which are physically embedded in the composite but cannot establish chemical bonds with the polymeric matrix.

The inability of unsilanized fillers for bonding with the polymeric matrix affects the development of the composite’s viscoelastic properties during polymerization^20^. The fact that the addition of unsilanized fillers can modify the mobility of the polymerizing resin inspired studies, which investigated this effect as a potential approach to reduce polymerization shrinkage stress^21,22^. These studies showed that nano-sized unsilanized silica fillers can reduce polymerization shrinkage stress of resin composites, with the extent of reduction being dependent on composite viscosity and the presence of other silanized fillers. The mechanisms of shrinkage stress reduction by virtue of unsilanized fillers include an improved flow capability at the unbonded filler/matrix interface, and the development of gaps around unsilanized fillers that act as internal sites for stress relief^23^. Because the detrimental effects of shrinkage stress (e.g. loss of marginal integrity, postoperative sensitivity, and secondary caries) are considered a major shortcoming of all resin composites^24^, efforts to reduce shrinkage stress are relevant for future material improvements.

Except the aforementioned studies on the incorporation of unsilanized non-soluble fillers as a means to reduce polymerization shrinkage stress in conventional composites^21,22^, the corresponding effect has not been investigated for unsilanized reactive fillers in bioactive composites. The present study addressed this issue by investigating the polymerization shrinkage behaviour of experimental composites functionalized with conventional BG 45S5 and an experimental low-sodium fluoride-containing BG. The aim of the study was to investigate the effect of varying amounts (0–60 wt%) of two BG types on linear shrinkage, degree of conversion (DC), shrinkage stress, and shrinkage stress kinetics. The null hypotheses assumed that these properties would not be affected by (I) the amount of unsilanized BG fillers, and (II) the BG type.

## Materials and methods

### Experimental resin composites

Experimental resin composites were prepared according to a previous study^12^. The resin matrix comprised bisphenol-A-glycidyldimethacrylate (Bis-GMA, Merck, Darmstadt, Germany) and triethylene glycol dimethacrylate (TEGDMA, Merck) in a weight ratio of 60:40. Camphorquinone (0.2 wt%; Merck) and ethyl-4-(dimethylamino) benzoate (0.8 wt%; Merck) were added to the blend of monomers. All components were mixed using a magnetic stirrer for 48 h.

BG 45S5 and inert barium glass were obtained as commercially available products from Schott (Mainz, Germany). The experimental low-sodium fluoride-containing BG was prepared on-request by Schott via the melt-quench route. In order to obtain similar particle sizes of BG 45S5 and the experimental BG, similar preparation and grinding procedures were used for both BG types. Inert barium glass fillers were silanized during the manufacturing process, whereas the BG fillers were unsilanized. The composition of BG and reinforcing (inert) fillers is shown in Table 1.

**Table 1:** Compositional details of bioactive glass and reinforcing fillers used in experimental composites.

	Bioactive glass 45S5	Experimental fluoride-containing bioactive glass	Inert barium glass
Particle size (d50)	3 µm	3 µm	1 µm
Composition (wt%)	45.0% SiO_2_ 24.5% CaO 24.5% Na_2_O 6.0% P_2_O_5_	33.5% SiO_2_ 33.0% CaO 10.5% Na_2_O 11.0% P_2_O_5_ 12.0% CaF_2_	55.0% SiO_2_ 25.0% BaO 10.0% Al_2_O_3_ 10.0% B_2_O_3_
Silanization (wt%)	None	None	3.2
Product name/LOT	G018-144/M111473	experimental batch	GM27884/Sil13696

Experimental composites with a total filler amount of 77 wt% were prepared by replacing a fraction of the reinforcing fillers (0–60 wt%) with either BG 45S5 or the experimental BG (Table 2). According to the type of BG, the experimental composite series were denoted as C-series (for conventional BG 45S5) and E-series (for the experimental BG). The control composite contained only reinforcing barium glass fillers.

**Table 2:** Composition of experimental composites.

Material designation	Filler composition (wt%)			Total filler amount (wt%)
	Bioactive glass 45S5	Experimental fluoride-containing bioactive glass	Reinforcing fillers (inert barium glass: silica = 2:1 by wt%)	
**Control**	0	0	77	77
**C-series**				
C-10	10	0	67	77
C-20	20	0	57	77
C-40	40	0	37	77
C-60	60	0	17	77
**E-series**				
E-10	0	10	67	77
E-20	0	20	57	77
E-40	0	40	37	77
E-60	0	60	17	77

The photoactivated resin system and the fillers were mixed using a dual asymmetric centrifugal mixing system (Speed Mixer TM DAC 150 FVZ, Hauschild & Co. KG, Hamm, Germany) at 2000 rpm. The mixing was performed in five 1-min intervals separated by 1-min breaks. After mixing, the prepared composites were deaerated in vacuum during 48 h.

### Linear shrinkage

Real-time linear shrinkage measurements were performed using a custom-made linometer, as described previously^25–28^. Briefly, the setup consisted of a thin aluminium platelet (12 × 12 mm, thickness = 0.25 mm, m = 0.4 g) loosely placed on a solid metal frame. A perpendicular diaphragm fixed to the bottom side of the platelet extended into a recess of the infrared measuring sensor. A discoid composite specimen of standardized volume (V = 42 mm^3^, d = 6 mm) was placed on the upper side of the aluminium platelet and flattened using a glass plate to a thickness of 1.5 mm. The light guide tip of the curing unit (Bluephase PowerCure, Ivoclar Vivadent, Schaan, Liechtenstein; curing unit tip diameter: 9 mm, emission wavelength range: 390–500 nm, radiant exitance: 1340 mW/cm^2^) was positioned directly above the glass plate, and the composite specimen was light-cured through the glass plate for 20 s, resulting in a total radiant exposure of 26.8 J/cm^2^. The curing unit performance was periodically verified using a calibrated and NIST-referenced UV–Vis spectrophotometer system (MARC; BlueLight Analytics, Halifax, Canada). Polymerization shrinkage caused the vertical displacement of the diaphragm which was detected by the infrared sensor at a data collection rate of 5 Hz and an accuracy of 0.1 µm. Linear shrinkage was measured in real-time for 15 min from the start of light-curing. The measurements were conducted inside a temperature-controlled chamber at 25 ± 1 °C, which simulated intraoral temperature after rubber-dam isolation^27^. The data were logged by a personal computer, using an analog-to-digital converter and custom-made software. Ten experimental runs were performed for each group (n = 10).

### Degree of conversion

The specimens used for linear shrinkage measurements were removed from the linometer immediately after completing the measurements, and mounted onto a diamond attenuated total reflectance (ATR) accessory of a Fourier transform infrared (FTIR) spectrometer (Cary 630 FTIR, Agilent Technologies, Santa Clara, CA, USA). The DC was evaluated on the bottom side of 1.5 mm thick discoid specimens, 15 min after the start of light-curing. This procedure of evaluating the DC at the endpoint of the observation period for linear shrinkage and shrinkage stress measurements was adopted from previous studies^25–27,29,30^.

The FTIR spectra were collected with 100 scans, using a resolution of 4 cm^−1^ in the wavelength range of 400–4000 cm^−1^. Spectra from uncured composites were recorded under the same conditions. Ten experimental runs were performed for each group (n = 10). DC was calculated from the changes in the ratio of absorbance intensities (peak heights) of aliphatic C=C (1638 cm^−1^) and aromatic C⋯C (1608 cm^−1^) spectral bands using the following equation^31^:

$$ {\text{DC}} (\% ) = \left( {1 - \frac{{(1638\; {\text{cm}}^{ - 1} /1608\;{\text{cm}}^{ - 1} )\;{\text{after curing}}}}{{(1638\;{\text{cm}}^{ - 1} /1608\;{\text{cm}}^{ - 1} )\;{\text{before curing}}}}} \right) \times 100 $$

### Shrinkage stress

Real-time measurements of shrinkage stress were performed using a custom-made stress analyser, as described previously^25–27,29,30^. The semi-rigid setup with compliance of 0.4 μm/N was used to simulate the partial relief of shrinkage stress through the displacement of dental hard tissues^27^. A standardized amount of the composite material (V = 42 mm^3^, d = 6 mm) was applied onto a metal cylinder connected to a load cell (PM 11-K; Mettler, Greifensee, Switzerland). The composite was flattened by a glass plate to a thickness of 1.5 mm in order to produce the discoid composite specimen with the base surface area of 28 mm^2^. Bonding the bases of the discoid specimens to the metal cylinder and the glass plate resulted in the ratio of bonded to unbonded surface area (C-factor) of 2.0. To ensure bonding of the composite to the glass plate and metal cylinder, their surfaces were sandblasted using aluminium oxide (50 μm; Renfert, Hilzingen, Germany), rinsed with a stream of demineralized water for 30 s, dried using pressurized air stream, and treated with a silane-containing primer Monobond Plus (Ivoclar Vivadent). To confirm that the sandblasted surfaces were clean from aluminium oxide particles, visual inspection was performed using a stereomicroscope at 40× magnification (M3Z; Leica/Wild, Heerbrugg, Switzerland). The composite specimen was light-cured through the glass plate in the same manner as described for the linear shrinkage measurements. Forces resulting from polymerization shrinkage were detected by the load cell at a data collection rate of 5 Hz and accuracy of 0.001 N. Real-time shrinkage stress data were collected for 15 min from the start of light-curing, inside a temperature-controlled chamber at 25 ± 1 °C. The data were logged by a personal computer, using an analog-to-digital converter and custom-made software. Ten experimental runs were performed for each group (n = 10). Shrinkage stress was calculated by dividing force values by the surface area of the composite specimen base (28 mm^2^). The obtained data were used to plot the curves of polymerization shrinkage stress versus time. First derivatives of these curves were calculated as a measure of the shrinkage stress rate. The plots of first derivatives versus time were used to estimate kinetic parameters: maximum shrinkage stress rate and time to achieve maximum shrinkage stress rate.

### Statistical analysis

Normality of distribution and homogeneity of variances were checked using Levene’s and Shapiro–Wilk’s tests, respectively. The effects of the BG type and BG amount were investigated by two-way ANOVA. After obtaining highly significant results (*p* < 0.001) for both factors and their interactions, mean values of linear shrinkage, DC, shrinkage stress, maximum shrinkage stress rate, and time to achieve maximum shrinkage stress rate were compared among the composites using one-way ANOVA and Tukey’s adjustment for multiple comparisons. The statistical analysis was performed using SPSS (version 20, IBM, Armonk, NY, USA) at an overall level of significance of α = 0.05.

## Results

**Figure 1:**
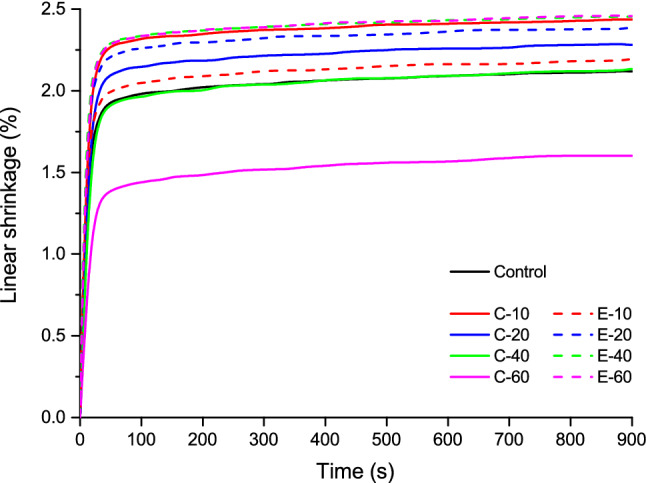
Averaged curves (n = 10) of linear shrinkage for the composites functionalized with bioactive glass 45S5 (continuous lines) and the experimental bioactive glass (dashed lines).

**Figure 2:**
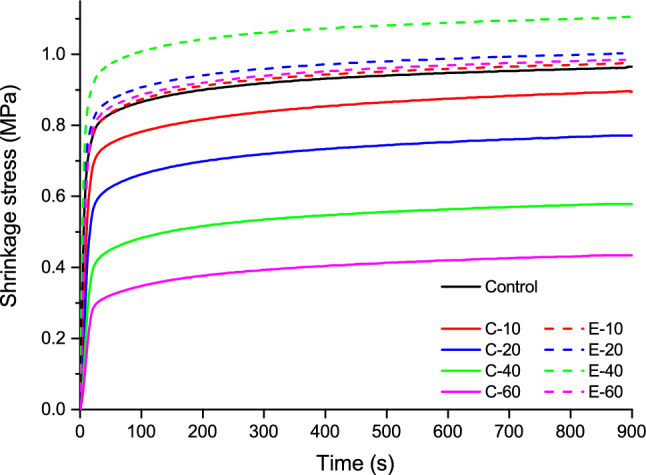
Averaged curves (n = 10) of shrinkage stress for the composites functionalized with bioactive glass 45S5 (continuous lines) and the experimental bioactive glass (dashed lines).

The real-time linear shrinkage and shrinkage stress data are illustrated in Figs. 1 and 2, respectively. Plots in Fig. 1 were used to determine linear shrinkage values at the end of the 15-min observation period, whereas the plots in Fig. 2 were used to determine the shrinkage stress at the end of the 15-min observation period, and the kinetic parameters: maximum shrinkage stress rate and time to achieve maximum shrinkage stress rate.

**Figure 3:**
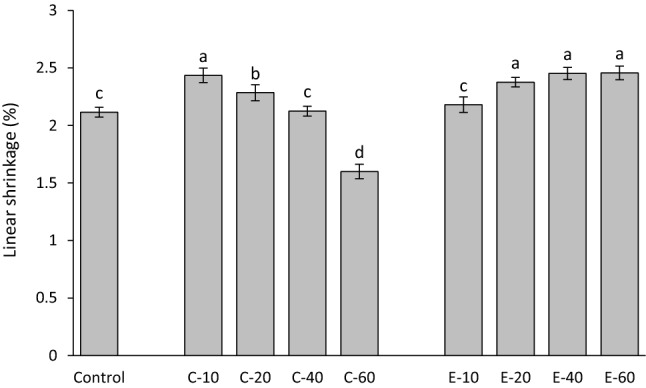
Linear shrinkage (mean values ± standard deviation) measured after 15 min. Same letters denote statistically homogeneous groups.

Linear shrinkage values measured 15 min after the start of light-curing are shown in Fig. 3. The addition of 10–20 wt% of BG 45S5 significantly increased linear shrinkage compared to the control composite. Within the C-series, a significant decrease in linear shrinkage was identified with increasing amounts of BG 45S5. The addition of 10 wt% of the experimental BG produced linear shrinkage similar to that of the control composite, whereas 20–60 wt% of the experimental BG significantly increased linear shrinkage.

**Figure 4:**
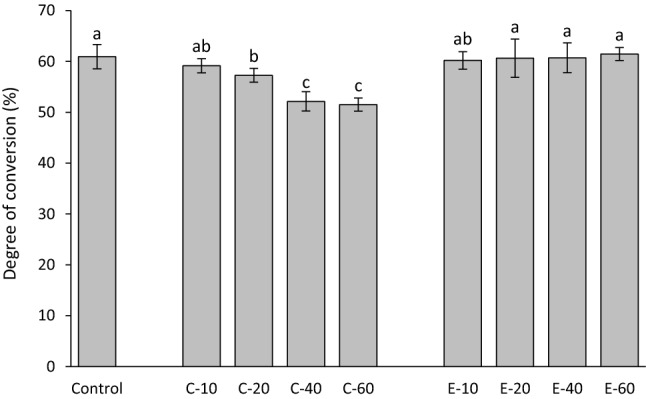
Degree of conversion (mean values ± standard deviation) measured after 15 min. Same letters denote statistically homogeneous groups.

The DC after 15 min of the start of light-curing is shown in Fig. 4. The composite with 10 wt% of BG 45S5 had similar DC as the control composite, whereas higher amounts of BG 45S5 led to significantly lower DC values. Within the C-series, an overall trend of DC decline with increasing amounts of BG 45S5 was observed. In contrast, the DC values of all composites in the E-series were statistically similar to that of the control composite.

**Figure 5:**
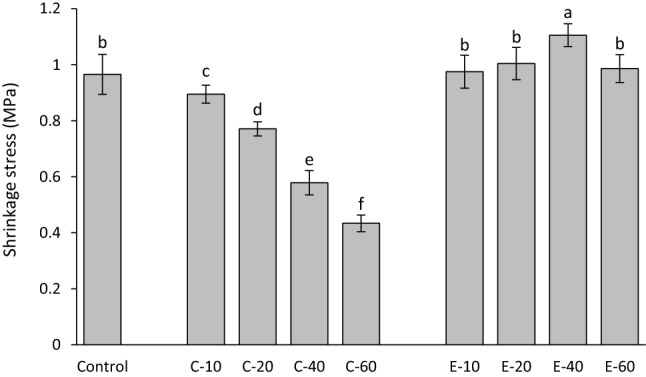
Shrinkage stress (mean values ± standard deviation) measured after 15 min. Same letters denote statistically homogeneous groups.

Shrinkage stress values measured 15 min after the start of light-curing are shown in Fig. 5. The addition of BG 45S5 resulted in a significant decline in shrinkage stress. In contrast, the addition of the experimental BG led to shrinkage stress values similar to that of the control composite, except for 40 wt% of the experimental BG, which produced a significantly higher shrinkage stress.

**Figure 6:**
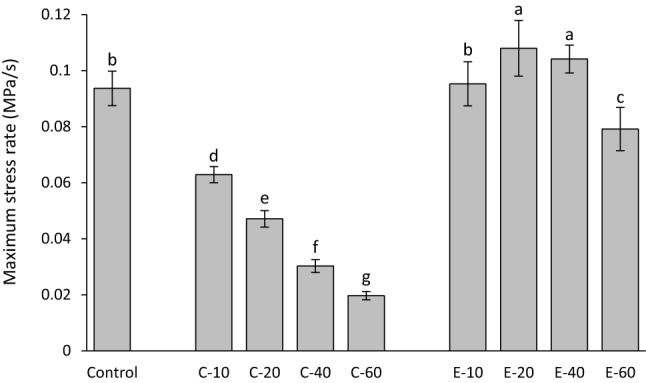
Maximum shrinkage stress rate (mean values ± standard deviation). Same letters denote statistically homogeneous groups.

Maximum shrinkage stress rates are shown in Fig. 6. The addition of BG 45S5 resulted in a significant decrease in maximum shrinkage stress rate. Compared to the control composite, the shrinkage stress rates for the composites functionalized with the experimental BG were unchanged (for 10 wt%), increased (for 20 and 40 wt%), or reduced (for 60 wt%).

**Figure 7:**
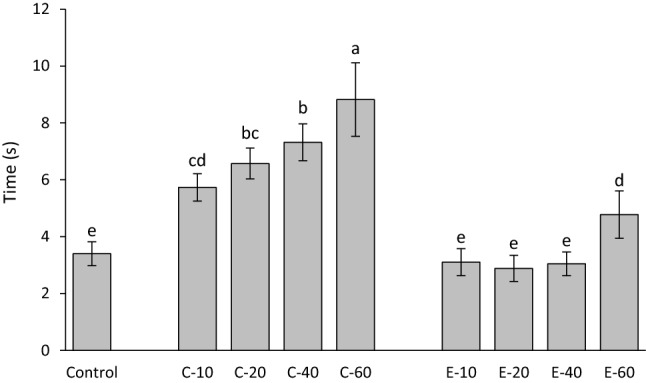
Time to achieve maximum shrinkage stress rate (mean values ± standard deviation). Same letters denote statistically homogeneous groups.

The time to achieve maximum shrinkage stress rate is shown in Fig. 7. The addition of BG 45S5 resulted in a significant increase in the time to achieve maximum shrinkage stress rate. The addition of 10–40 wt% of the experimental BG had no effect on time to achieve maximum shrinkage stress rate, whereas a significantly longer time to achieve maximum shrinkage stress rate was observed for 60 wt% of the experimental BG.

## Discussion

This study investigated the effect of replacing silanized reinforcing fillers with 0–60 wt% of different unsilanized BG fillers on shrinkage properties of experimental composites. Although the composites with high amounts of unsilanized BG fillers would not be beneficial clinically due to their poor mechanical properties^13^, the wide range of BG amounts was chosen in order to explore shrinkage behaviour as a function of the wt% of unsilanized BG fillers^22^. The first null hypothesis was accepted only for the DC of the composites functionalized with the experimental BG, which was not affected by the BG amount. As the BG amount and type significantly influenced linear shrinkage, shrinkage stress, maximum shrinkage rate, and time to achieve maximum shrinkage rate, both null hypotheses were rejected for these properties.

The decrease of DC with increasing BG amounts observed in the composites functionalized with BG 45S5 is consistent with previous studies of experimental composites featuring similar compositions^32,33^. On the other hand, no negative effect on DC was identified in composites functionalized with the experimental low-sodium fluoride-containing BG in a previous study^12^ nor in the present study. The potential of BG 45S5 for inhibiting methacrylate polymerization and limiting the final DC has been tackled in previous studies^12,32–35^, and the polymerization inhibition was explained by the inactivation of free radicals mediated by oxides on the surface of unsilanized BG particles^36^. A detailed analysis of the mechanisms responsible for different effects of BG 45S5 and the experimental BG on polymerization reaction and final DC values is out of the scope of this study. Nevertheless, the significantly different influence of the two types of BG fillers on the DC of experimental composites enabled insight into shrinkage behaviour that was either governed by the changing DC (C-series) or that was DC-independent (E-series). As DC has been shown to be diminished at the boundary layer of resin that is immediately adjacent to filler particles, incorporating larger particles reduces total filler surface area and thus can be expected to improve DC^37^. This improvement in DC was not identified in our study when smaller reinforcing filler particles (d50 = 1 µm) were replaced by larger BG fillers (d50 = 3 µm). Considering that BG fillers had a wide particle size distribution, it is likely that the nominal difference in d50 was too small to exert a measurable effect on DC. Even though the DC in the E-series was not affected by the presence of BG fillers, the BG amounts of 20 wt% and higher led to a significant increase in linear shrinkage. Unlike volumetric shrinkage, which is expected to increase proportionally to DC^20^, linear shrinkage can deviate from being directly dependent on DC due to the tendency of linometers to measure only the portion of shrinkage that takes place after gelation^38^. Before the onset of gelation, the reduction in the volume of the discoid specimen can be partly compensated by the shrinkage in the radial direction^39^. At the gel point, the polymerizing composite reaches the elastic modulus that is sufficient for overcoming the gravitational force of the aluminium platelet (0.004 N) and producing a measurable displacement^40^. Therefore, the development of the composite’s viscoelastic properties during polymerization determines the fraction of volumetric shrinkage that is captured by linear measurements. As the viscoelastic properties of the experimental composites in this study were affected by variations in relative amounts of silanized and unsilanized fillers^21^, different fractions of total volumetric shrinkage were captured by linear measurements. Because the development of shrinkage stress is primarily governed by post-gel shrinkage, the obtained linear shrinkage values are meaningful regardless of not being directly correlated to the overall volumetric shrinkage.

In analogy to the E-series, an increase in linear shrinkage caused by the addition of BG fillers was also identified in the C-series for BG amounts of 10–20 wt% (compared to the control composite). For higher BG amounts (40–60 wt%), that effect was surpassed by a significant decrease in DC, leading to the overall lower linear shrinkage. Considering the results from the C-series and E-series together, it appears that the addition of unsilanized BG fillers increased linear shrinkage unless the DC was diminished due to high BG amounts. In the latter case, the effect of diminished DC prevailed, leading to an overall decrease in linear shrinkage.

The trend of DC being decreased by increasing BG amounts appears to be the major determinant of shrinkage stress behaviour in the C-series. As DC was gradually reduced throughout the C-series, the shrinkage stress parameters were affected accordingly: shrinkage stress after 15 min and maximum shrinkage stress rate were decreased, whereas the time to achieve maximum shrinkage rate was prolonged. An exception to this pattern was identified for high amounts of BG 45S5 (40–60 wt%) for which the DC values reached a plateau, whereas the trend of decreasing shrinkage stress and maximum stress rate continued. Variations in the BG amount thus produced a comparatively more pronounced influence on shrinkage stress parameters than on DC. Nevertheless, even small, non-significant DC differences can lead to significant differences in elastic moduli^41^, which in turn reflect on shrinkage stress. This might explain why statistically significant differences in the shrinkage stress properties were observed throughout the whole C-series despite the corresponding DC values plateauing for BG amounts of 40–60 wt%.

The fact that the DC in the E-series remained unchanged by varying BG amounts helped to isolate the direct effect of unsilanized BG fillers on shrinkage stress development without the influence of DC as a confounding factor. Contrary to the expectations derived from previous studies that increasing amounts of unsilanized BG fillers would diminish shrinkage stress^21,22^, such an effect was not observed. Moreover, a small but statistically significant increase in shrinkage stress and maximum stress rate was identified for the composites with intermediate BG amounts (E-40 for shrinkage stress; E-20 and E-40 for maximum stress rate). Although being surprising, the results of shrinkage stress peaking for a certain BG amount and decreasing for lower or higher BG amounts are in line with a report which showed that the addition of unsilanized fillers does not necessarily affect shrinkage stress in a mathematically monotonic manner^22^. The shrinkage stress development is determined by a complex interaction of the composite’s viscoelastic properties and polymerization kinetics. Both of these factors are independently affected by varying proportions of silanized and unsilanized fillers^21,42^. Additionally, the interactions of unsilanized fillers with monomers can affect molecular mobility at the filler/resin interface, thereby counteracting the expected reduction in shrinkage stress^21^. These interactions produced a non-monotonic dependence of shrinkage stress properties on the amount of BG fillers, whereby shrinkage stress and maximum stress rate reached peak values for certain BG amounts (40 and 20 wt%, respectively), and declined afterward. On the other hand, the time to achieve maximum shrinkage rate was constant for BG amounts of up to 40 wt% and then increased for 60 wt% of BG.

The aforementioned small, but statistically significant, increase in polymerization shrinkage stress and stress rate identified in the E-series was speculated to originate from the effect of unsilanized BG particles on the development of viscoelastic properties during polymerization. A detailed rheokinetic analysis was not performed in the present study because such measurements are inherently challenging^43,44^, and the hypothesized effect of unsilanized particles on stress reduction was not expected to be mediated by changes in rheological behaviour. Instead, the stress reduction was expected to occur due to the formation of gaps at the filler/resin interface^23^, and consequently lower interfacial stress concentration^22^. As the stress reduction was not identified in the E-series, and the addition of unsilanized fillers has been previously reported to increase the viscosity of experimental composites^21^, the effect of unsilanized fillers on rheological properties was speculated to have caused the small increase in polymerization shrinkage stress and stress rate in the E-series. Another factor that might have affected the rheological properties of the experimental composites is the difference in particle size between the reinforcing and BG fillers. However, considering that the grinding processing of glass fillers produced wide particle size distributions, the nominal differences in particle size likely had a negligible impact on the obtained results.

The effect of the unsilanized BG fillers on the reduction of shrinkage stress was identified only as a consequence of the reduction in DC (C-series) but not when DC was maintained (E-series). Except the significantly slower development of shrinkage stress in the composite with the highest BG amount (E-60), no direct DC-independent reduction of shrinkage stress was produced by the unsilanized BG fillers. The discrepancy between these results and the results of the previous studies that identified a stress-reducing potential of unsilanized fillers^21,22^ can be attributed to two major differences in experimental conditions. First, the mentioned studies used nano-sized silica particles (average size of 40 nm), whereas the BG particles used in the present study were two orders of magnitude larger. A considerable difference in the interfacial surface area between the resin and unsilanized filler particles could have affected their potential to relieve shrinkage stress. Second, shrinkage stress in the mentioned studies was measured using a rigid device with a feedback loop that compensated for load cell compliance. The stress values obtained by this type of devices are more sensitive to the composite’s elastic modulus compared to the stress values obtained by a more compliant device used in the present study^45^. It is therefore plausible that the results of the mentioned studies^21,22^ were governed by the effect of unsilanized fillers on the development of composite’s elastic modulus during polymerization, whereas our results were more dependent on the effect of the unsilanized BG fillers on the resin mobility. Considering the major role of device compliance in the measurements of shrinkage stress, the results obtained using compliance similar to that of dental hard tissues in this study can be regarded as more clinically meaningful than those obtained in highly rigid setups^46^.

Considering the negative effects of unsilanized BG fillers on the mechanical properties of resin composites^13,14^, it should be noted that the clinical advantage of the shrinkage stress relief identified in the C-series may be limited. Although considerable stress reductions were observed in the C-series by using BG amounts of 40–60 wt%, such high fractions of unsilanized versus reinforcing fillers would considerably diminish mechanical properties, especially since higher BG amounts also led to reduced DC^47^. The mechanical properties could be expected to further decrease over time due to high water sorption caused by soluble BG fillers^48^.

The curing conditions used in this study were determined in a preliminary study, which showed that curing with 1340 mW/cm^2^ for 20 s produced homogeneous curing throughout the 1.5 mm thick specimens. Although the specimens were thoroughly cured, the development of polymerization shrinkage stress might have been influenced by the effect of unsilanized BG fillers on polymerization kinetics^33^. This aspect will be addressed in forthcoming studies that will investigate the dynamic changes of DC and light transmittance during polymerization using real-time FTIR and UV–vis spectrometry, respectively.

## Conclusions

The effect of unsilanized bioactive glass fillers on polymerization shrinkage properties differed between two types of bioactive glass. The diminishing effect of bioactive glass 45S5 on shrinkage stress was mediated by a decrease in degree of conversion caused by this bioactive glass type. In contrast, no reduction of shrinkage stress was observed for the experimental low-sodium fluoride-containing bioactive glass. Therefore, the hypothesized benefits of unsilanized bioactive glass fillers on the reduction of polymerization shrinkage stress were not identified under the conditions of this study, unless this reduction was attained indirectly through a diminished degree of conversion.

## Data Availability

The datasets generated during and/or analysed during the current study are available from the corresponding author on reasonable request.

## References

[1] Nedeljkovic I (2020). Secondary caries: prevalence, characteristics, and approach. Clin. Oral Investig..

[2] O’Donnell JNR, Langhorst SE, Fow MD, Skrtic D, Antonucci JM (2008). Light-cured dimethacrylate-based resins and their composites: comparative study of mechanical strength, water sorption, and ion release. J. Bioact. Compat. Polym..

[3] Beyth N, Yudovin-Farber I, Bahir R, Domb AJ, Weiss EI (2006). Antibacterial activity of dental composites containing quaternary ammonium polyethylenimine nanoparticles against *Streptococcus mutans*. Biomaterials.

[4] Odermatt R (2020). Bioactivity and physico-chemical properties of dental composites functionalized with nano- vs. micro-sized bioactive glass. J. Clin. Med..

[5] Al-eesa NA, Johal A, Hill RG, Wong FSL (2018). Fluoride containing bioactive glass composite for orthodontic adhesives—apatite formation properties. Dent. Mater..

[6] Rizk M (2020). Mineral precipitation, polymerization properties and bonding performance of universal dental adhesives doped with polyhedral oligomeric silsesquioxanes. Int. J. Adhes. Adhes..

[7] Al-eesa NA, Wong FSL, Johal A, Hill RG (2017). Fluoride containing bioactive glass composite for orthodontic adhesives—ion release properties. Dent. Mater..

[8] Tauböck TT (2014). Functionalizing a dentin bonding resin to become bioactive. Dent. Mater..

[9] Tezvergil-Mutluay A (2017). Effects of composites containing bioactive glasses on demineralized dentin. J. Dent. Res..

[10] Khvostenko D, Hilton TJ, Ferracane JL, Mitchell JC, Kruzic JJ (2016). Bioactive glass fillers reduce bacterial penetration into marginal gaps for composite restorations. Dent. Mater..

[11] Davis HB, Gwinner F, Mitchell JC, Ferracane JL (2014). Ion release from, and fluoride recharge of a composite with a fluoride-containing bioactive glass. Dent. Mater..

[12] Par M, Attin T, Tarle Z, Tauböck TT (2020). A new customized bioactive glass filler to functionalize resin composites: acid-neutralizing capability, degree of conversion, and apatite precipitation. J. Clin. Med..

[13] Par M, Tarle Z, Hickel R, Ilie N (2019). Mechanical properties of experimental composites containing bioactive glass after artificial aging in water and ethanol. Clin. Oral Investig..

[14] Par M, Tarle Z, Hickel R, Ilie N (2018). Dentin bond strength of experimental composites containing bioactive glass: changes during aging for up to 1 year. J. Adhes. Dent..

[15] Khvostenko D, Mitchell JC, Hilton TJ, Ferracane JL, Kruzic JJ (2013). Mechanical performance of novel bioactive glass containing dental restorative composites. Dent. Mater..

[16] Antonucci JM, Dickens SH, Fowler BO, Xu HHK (2005). Chemistry of silanes: interfaces in dental polymers and composites. J. Res. Natl. Inst. Stand. Technol..

[17] Chan KS (2007). Improving fracture toughness of dental nanocomposites by interface engineering and micromechanics. Eng. Fract. Mech..

[18] Dieckmann P, Mohn D, Zehnder M, Attin T, Tauböck TT (2019). Light transmittance and polymerization of bulk-fill composite materials doped with bioactive micro-fillers. Materials.

[19] Oral O, Lassila LV, Kumbuloglu O, Vallittu PK (2014). Bioactive glass particulate filler composite: effect of coupling of fillers and filler loading on some physical properties. Dent. Mater..

[20] Braga R, Ballester R, Ferracane J (2005). Factors involved in the development of polymerization shrinkage stress in resin-composites: a systematic review. Dent. Mater..

[21] Condon JR, Ferracane JL (1998). Reduction of composite contraction stress through non-bonded microfiller particles. Dent. Mater..

[22] Condon JR, Ferracane JL (2002). Reduced polymerization stress through non-bonded nanofiller particles. Biomaterials.

[23] Feng L, Suh BI, Shortall AC (2010). Formation of gaps at the filler–resin interface induced by polymerization contraction stress. Dent. Mater..

[24] Ferracane JL (2008). Placing dental composites—a stressful experience. Oper. Dent..

[25] Tauböck TT, Bortolotto T, Buchalla W, Attin T, Krejci I (2010). Influence of light-curing protocols on polymerization shrinkage and shrinkage force of a dual-cured core build-up resin composite: shrinkage and force of dual-cured composite. Eur. J. Oral Sci..

[26] Tauböck TT (2014). Effect of modulated photo-activation on polymerization shrinkage behavior of dental restorative resin composites. Eur. J. Oral Sci..

[27] Tauböck TT, Jäger F, Attin T (2019). Polymerization shrinkage and shrinkage force kinetics of high- and low-viscosity dimethacrylate- and ormocer-based bulk-fill resin composites. Odontology.

[28] Lottanti S, Tauböck TT, Zehnder M (2014). Shrinkage of backfill gutta-percha upon cooling. J. Endod..

[29] Tauböck TT, Tarle Z, Marovic D, Attin T (2015). Pre-heating of high-viscosity bulk-fill resin composites: effects on shrinkage force and monomer conversion. J. Dent..

[30] Marovic D, Tauböck TT, Attin T, Panduric V, Tarle Z (2015). Monomer conversion and shrinkage force kinetics of low-viscosity bulk-fill resin composites. Acta Odontol. Scand..

[31] Rueggeberg FA, Hashinger DT, Fairhurst CW (1990). Calibration of FTIR conversion analysis of contemporary dental resin composites. Dent. Mater..

[32] Par M (2018). Curing potential of experimental resin composites with systematically varying amount of bioactive glass: degree of conversion, light transmittance and depth of cure. J. Dent..

[33] Par M, Tarle Z, Hickel R, Ilie N (2018). Polymerization kinetics of experimental bioactive composites containing bioactive glass. J. Dent..

[34] Par M (2020). Curing potential of experimental resin composites filled with bioactive glass: a comparison between Bis-EMA and UDMA based resin systems. Dent. Mater..

[35] Par M, Spanovic N, Tauböck TT, Attin T, Tarle Z (2019). Degree of conversion of experimental resin composites containing bioactive glass 45S5: the effect of post-cure heating. Sci. Rep..

[36] Plueddemann EP, Deanin RD, Schott NR (1974). Catalytic effects in bonding thermosetting resins to silane-treated fillers. Fillers and Reinforcements for Plastics, 134.

[37] Sirovica S (2020). Origin of micro-scale heterogeneity in polymerisation of photo-activated resin composites. Nat. Commun..

[38] Sakaguchi R (2004). Critical configuration analysis of four methods for measuring polymerization shrinkage strain of composites. Dent. Mater..

[39] Lee I-B, Cho B-H, Son H-H, Um C-M, Lim B-S (2006). The effect of consistency, specimen geometry and adhesion on the axial polymerization shrinkage measurement of light cured composites. Dent. Mater..

[40] de Gee AJ, Feilzer AJ, Davidson CL (1993). True linear polymerization shrinkage of unfilled resins and composites determined with a linometer. Dent. Mater..

[41] Lovell LG, Berchtold KA, Elliott JE, Lu H, Bowman CN (2001). Understanding the kinetics and network formation of dimethacrylate dental resins. Polym. Adv. Technol..

[42] Par M, Tarle Z, Hickel R, Ilie N (2018). Real-time curing characteristics of experimental resin composites containing amorphous calcium phosphate. Eur. J. Oral Sci..

[43] Macosko CW (1985). Rheological changes during crosslinking. Br. Polym. J..

[44] Malkin AY (2009). The state of the art in the rheology of polymers: Achievements and challenges. Polym. Sci. Ser. A.

[45] Wang Z, Chiang MYM (2016). System compliance dictates the effect of composite filler content on polymerization shrinkage stress. Dent. Mater..

[46] Lee SH, Chang J, Ferracane J, Lee IB (2007). Influence of instrument compliance and specimen thickness on the polymerization shrinkage stress measurement of light-cured composites. Dent. Mater..

[47] Calheiros FC, Daronch M, Rueggeberg FA, Braga RR (2008). Degree of conversion and mechanical properties of a BisGMA:TEGDMA composite as a function of the applied radiant exposure. J. Biomed. Mater. Res. B Appl. Biomater..

[48] Par M (2019). Long-term water sorption and solubility of experimental bioactive composites based on amorphous calcium phosphate and bioactive glass. Dent. Mater. J..

